# Transcriptional Responses of *Arabidopsis thaliana* during Wilt Disease Caused by the Soil-Borne Phytopathogenic Bacterium, *Ralstonia solanacearum*

**DOI:** 10.1371/journal.pone.0002589

**Published:** 2008-07-02

**Authors:** Jian Hu, Xavier Barlet, Laurent Deslandes, Judith Hirsch, Dong Xin Feng, Imre Somssich, Yves Marco

**Affiliations:** 1 Laboratoire des Interactions Plantes-Microorganismes (LIPM), UMR CNRS/INRA 2594/441, Castanet-Tolosan, France; 2 Department of Biochemistry and Molecular Biology, College of Biological Sciences, China Agricultural University, Beijing, China; 3 Interactions Riz-parasites, UMR BGPI CIRAD TA A-54/K, Campus International de Baillarguet, Montpellier, France; 4 Department of International Cooperation, Chinese Academy of Agricultural Sciences, Beijing, People's Republic of China; 5 Max Planck Institut für Züchtungsforschung, Abteilung Biochemie, Cologne, Germany; University of Melbourne, Australia

## Abstract

Bacterial wilt is a common disease that causes severe yield and quality losses in many plants. In the present study, we used the model *Ralstonia solanacearum*-*Arabidopsis thaliana* pathosystem to study transcriptional changes associated with wilt disease development. Susceptible Col-5 plants and *RRS1-R*-containing resistant Nd-1 plants were root-inoculated with *R. solanacearum* strains harbouring or lacking the matching *PopP2* avirulence gene. Gene expression was marginally affected in leaves during the early stages of infection. Major changes in transcript levels took place between 4 and 5 days after pathogen inoculation, at the onset of appearance of wilt symptoms. Up-regulated genes in diseased plants included ABA-, senescence- and basal resistance-associated genes. The influence of the plant genetic background on disease-associated gene expression is weak although some genes appeared to be specifically up-regulated in Nd-1 plants. Inactivation of some disease-associated genes led to alterations in the plant responses to a virulent strain of the pathogen. In contrast to other pathosystems, very little overlap in gene expression was detected between the early phases of the resistance response and the late stages of disease development. This observation may be explained by the fact that above-ground tissues were sampled for profiling whereas the bacteria were applied to root tissues.

This exhaustive analysis of Arabidopsis genes whose expression is modulated during bacterial wilt development paves the way for dissecting plant networks activated by recognition of *R. solanacearum* effectors in susceptible plants.

## Introduction

To combat pathogenic microbes, plants have evolved a complex network of synergistic defensive strategies, termed basal defense or non-host resistance. Perception at the cell surface of Pathogen-Associated Molecular Patterns (PAMPs) of a microorganism leads to the activation of elaborate plant basal defenses often sufficient to resist most pathogens [1], [2]. Some microbes can however suppress basal defense but then face a stronger and more specialized line of defense based on *R*-gene mediated resistance. Recognition events between plant *R* and microbial *avirulence (avr)* gene products initiate a transcriptional reprogramming resulting ultimately in a defense response often associated with the hypersensitive response (HR) [3], [4], a localized cell death at the site of pathogen inoculation. In addition, plant responses to some pathogens can lead to systemic acquired resistance, which immunizes against subsequent infections. Endogenous signal molecules such as salicylic acid (SA) play a key role in signalling for this type of resistance [5].

In absence of a specific perception by the host plant, invading microorganisms multiply and spread within the plant, leading to disease and eventually to death of the infected host. In this type of interaction termed compatible, between a susceptible plant and a virulent pathogen, the plant defense system is activated to a certain extent but confers only a variable level of resistance. It is currently assumed that the plant signal transduction mechanisms are largely shared between compatible and incompatible interactions. A broad range of defense responses in the early phases of the resistant response are indeed very similar to those in late compatible interactions [6], [7]. This assumption is however based on a limited number of studies of interactions often resulting in an HR.

Plant infection and colonization by bacterial pathogens require effector molecules delivered into the plant by a type III secretion system encoded by the so-called hypersensitive response and pathogenicity bacterial gene cluster (*hrp*), required both for HR and disease development [8], [9]. Some of these effector proteins have been identified as *avr* factors recognized by the corresponding *R* gene products [10]. Their role remains generally poorly understood although some of them play crucial roles in virulence by suppressing/modulating plant defense responses allowing bacterial multiplication and spreading [11].

*Ralstonia solanacearum* is a Gram-negative soil-borne β-proteobacterium that causes bacterial wilt disease in diverse and important food crops such as tomato, potato, banana and ginger [12]. In tomato where disease development has been well studied, bacteria attach to root surfaces and form micro-colonies, especially at the root elongation zone and sites of lateral root emergence. They subsequently invade the intercellular spaces of the root cortex and, after a few days, colonize the intercellular spaces of the inner cortex and vascular parenchyma. After penetration of the xylem vessels, bacteria spread rapidly to the aerial parts of the infected plants. Complete wilting of the host, probably caused in part by this extensive colonization and a high exopolysaccharide production in xylem vessels, is observed 5 to 8 days after inoculation [13]. The *R. solanacearum* genome sequence recently allowed the identification of approximately 80 putative effectors whose targets in the plant cell and their roles during infection remain to be elucidated [14], [15], [16].

The genetic determinants for resistance to *R. solanacearum* are complex and still poorly characterized, except in *Arabidopsis thaliana* in which a gene, *RRS1-R*, identified in the resistant Nd-1 accession, was shown to confer a broad spectrum resistance to this root pathogen [17]. The matching Avr protein, PopP2, a putative cysteine protease belonging to the YopJ/AvrXv family, physically interacts with the R protein [18]. RRS1-R is a NBS-LRR-type protein containing an additional DNA binding domain [17].

Very little is known on the transcriptional changes induced by *R. solanacearum* in Arabidopsis and other plants. SA, an endogenous signal molecule playing a key role in resistance to many pathogens, appears to have a minor effect on the *RRS1-R*-mediated resistance and is not required for basal resistance to bacterial wilt [19], [17]. In contrast, a delay in symptom appearance was observed in *ein2-1* plants altered in ethylene signalling, and also a phenotypic suppressor of *abi1-1*, an abscissic acid (ABA) insensitive mutant [19]. A direct involvement of ABA signalling in the control of Arabidopsis resistance to *R. solanacearum* is supported by the enhanced susceptibility of *abi1-1* and *abi2-1*, two ABA-insensitive mutants [20]. The constitutive expression of some ABA signalling regulators, including *ABI1-1* and *ABI2-1* in the *irx* mutants affecting CESA proteins and exhibiting an enhanced resistance to *R. solanacearum*, also implicates this phytohormone in wilt disease development [20].

Knowledge of the events involved in disease development is based on a limited number of studies and very little is known on wilt diseases. Here, whole genome expression analysis was conducted on resistant and susceptible Arabidopsis accessions challenged with *R. solanacearum*. Two disease situations were considered: Col-5 plants inoculated with the virulent GMI1000 strain, as well as Nd-1 plants challenged with the same bacterial strain deleted of the *avr PopP2* gene, in both cases developing wilt disease symptoms. In contrast, Nd-1 plants challenged with GMI1000 are fully resistant to the pathogen. The bacteria were root-inoculated and leaf tissues were sampled for microarray analyses. Disease-associated genes were identified in both different genetic backgrounds and a reverse genetic approach for some of these genes was used to identify Arabidopsis genes whose inactivation led to an altered phenotype in response to *R. solanacearum*. This work constitutes the first exhaustive study of the molecular mechanisms associated with bacterial wilt disease development.

## Results

### Identification of Arabidopsis genes activated during wilt disease development

General changes in gene expression in resistant and susceptible plants to *R. solanacearum* were identified by microarray analysis using the ATH1 Affymetrix gene chips. Nd-1 plants are resistant to the GMI1000 *R. solanacearum* strain expressing the *avr PopP2* gene, but are fully susceptible to the same strain deleted of *PopP2* (GMI1000ΔPopP2) [17]. Col-5 plants that do not possess the *RRS1-R* gene are fully susceptible to GMI1000. No differences are detectable in the kinetics or intensity of wilt symptoms developed between diseased Nd-1 and Col-5 plants. In contrast to other pathosystems where resistance is associated with HR development, no visible symptom accompanies the *RRS1-R*-mediated resistance. To gain some insight on the transcriptional reprogramming occurring during the different types of interactions, bacterial strains differing only by the presence of *PopP2* were used to inoculate Nd-1 and Col-5 plants. Samples were harvested at the time of inoculation, at 6, 12, 24 hours thereafter at which times no symptoms were visible, 5 days after inoculation when the first wilt symptoms became visible (25% of wilted leaves: disease index 1, D1), and finally 8 days after inoculation when 75% of leaves were completely wilted (disease index 3, D3). Early time points were chosen assuming that early changes in gene expression in resistant plants should occur within the first 24 hours. In order to identify genes associated with wilt disease, we compared the global gene expression patterns in Nd-1 and Col-5 diseased plants to those of symptomless Nd-1 resistant plants.

**Figure 1:**
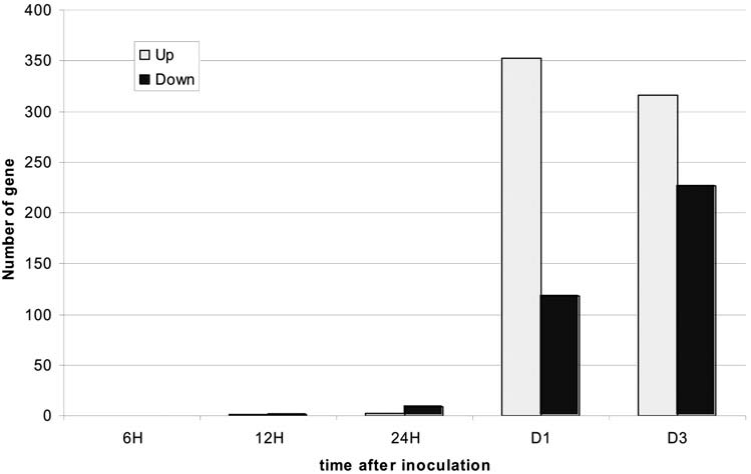
Diagram showing the number of up- and down-regulated genes (in white and black, respectively) identified by microarray analysis in both compatible interactions (Col-0/GMI1000 and Nd-1/GMI1000ΔPopP2) versus the incompatible interaction (Nd-1/GMI1000) at different times after pathogen inoculation. Up-regulated genes were selected using Signal Log_2_ Ratio ≥1, and down-regulated genes using a Signal Log_2_ Ratio ≤−1, using a corrected *p-value* ≤0.05 for both classes of genes.

**Figure 2:**
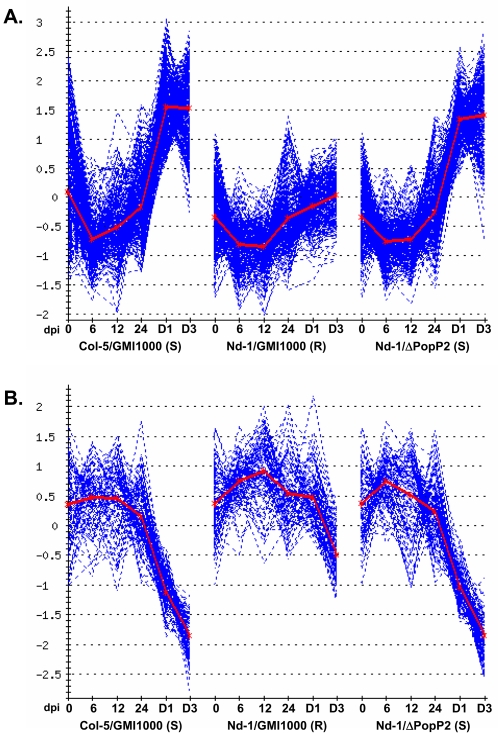
Cluster analysis of disease-associated genes was performed using Adap_Cluster (MIN_NR_Genes = 2, s = 0.95) [44]. A. Diagram corresponding to 305 disease-associated up-regulated genes. B. Diagram corresponding to 99 disease-associated down-regulated genes. Each diagram presents the normalized expression profiles of the genes (blue lines) and the mean expression profile is shown in red. S, Susceptible; R, Resistant.

Very few genes were differentially expressed in the early phases (6, 12 and 24 hours post inoculation) of the interactions (Figure 1 and Table S1). This observation may be explained by the fact that, at these stages, sampled aerial tissues were not directly in contact with the root-inoculated bacteria. Due to the low differential expression levels of these genes in the different interactions, these data could not be validated by quantitative real time PCR (Q-RT-PCR; Figure 1). At the onset of wilt symptom appearance, a massive shift in gene expression was detected. Using a cut off for differential expression of 2 (Signal Log_2_ Ratio of 1) and a corrected *p-value* ≤0.05, we identified 353 up- and 118 down-regulated genes in the Nd-1 and Col-5 diseased plants compared to Nd-1 resistant plants, 5 days after inoculation. The complete lists of these genes can be found in Table S2 and S3. Most up-regulated disease-associated genes were activated between 1 and 5 days after inoculation and remained generally expressed at high levels until death of the plants (Figure 2A). A majority of down-regulated genes in diseased plants were genes whose expression levels decreased strongly in wilting plants but remained unaffected in healthy resistant plants (Figure 2B).

**Figure 3:**
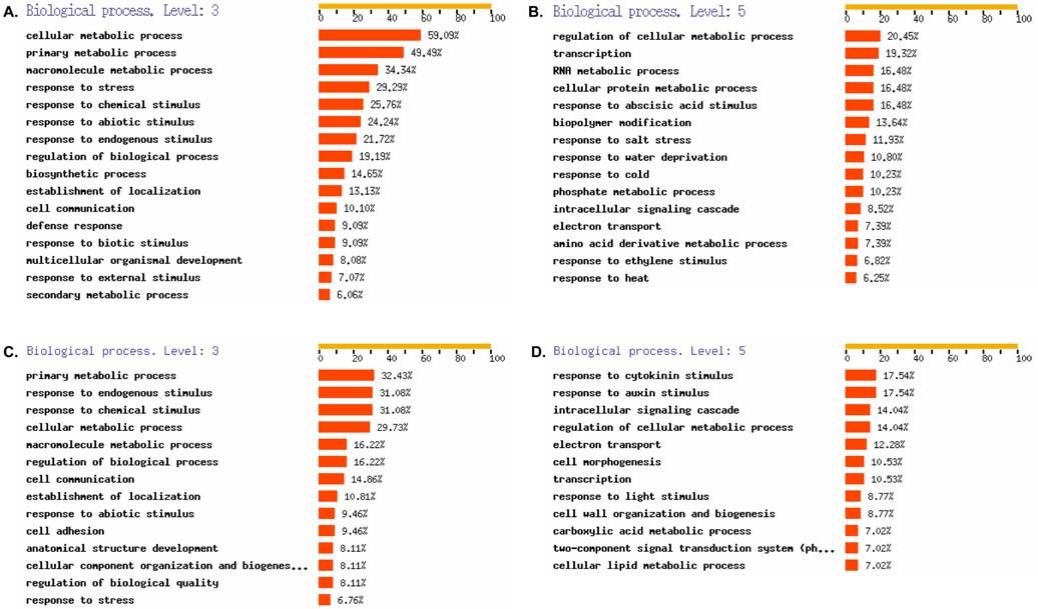
GO categorization of Arabidopsis genes differentially expressed after inoculation with *R. solanacearum* using FatiGo [42]. A. and B. 198 genes with annotations out of 352 disease-associated total up-regulated genes categorized at GO biological processes level 3 and 5, respectively. C. and D. 74 genes with annotations out of 118 total disease-associated down-regulated genes, categorized at biological processes level 3 and level 5. Percentages relate to the total number of genes with an ontology at each level. Only categories corresponding to more than 6% of total number of genes are shown.

The up-regulated genes are involved in various metabolic processes, signal transduction, transcriptional regulation and responses to various stresses (Figure 3A and 3B). Among the disease down-regulated genes, a significant number of genes associated to various developmental processes were identified and included a relatively high proportion of genes linked with auxin and cytokinin signalling pathways, suggesting that normal developmental processes are strongly affected in wilting plants (Figure 3D). Altogether, these data illustrate the complexity of the processes associated to disease development.

### ABA- Senescence- and basal defense-related genes were differentially expressed during wilt disease development

Recent studies suggested that ABA plays an important role in bacterial wilt disease development [20]. A recent microarray analysis obtained from Arabidopsis seedlings treated with this phytohormone led to the identification of ABA-regulated transcription networks [21]. By comparing these data with our list of genes, we could establish that 40% of the genes up-regulated during wilt disease development were involved in the biosynthesis and signalling of this phytohormone (Table S4A) [21]. Among them, *NCED3* (At3g14440) encoding a 9-cis-epoxycarotenoid dioxygenase, a key regulatory enzyme in ABA biosynthesis, genes encoding negative regulators of the ABA-related signalling such as PP2C *ABI1* (At4g26080), *ABI2* (At5g57050) and *P2C-HAB1* (At1g72770), and transcription factors (e.g. *ATHB-7*-At2g46680 and *ATHB-12*-At3g61890), as well as ABA marker genes such as *RAB18, RD22, and RD29A* were identified. The water stress provoked by the massive invasion of xylem vessels by bacteria may be one of the causes of the massive activation of ABA-related genes during disease.

Our data confirmed previous studies showing that pathogen infection induces the expression of senescence-associated genes (Table S4B) [22], [23], [24]. Indeed, 132 of the 353 disease up-regulated genes (37%) corresponded to genes differentially expressed in senescing plants.

Among the disease-associated genes, only very few SA-associated genes were found, in agreement with previous observations indicating that this signal molecule does not play a major role in *RRS1-R*-mediated-resistance as well as in wilt disease development [17], [19].

Additionally, a high proportion of disease up-regulated genes (46%–136/353) were genes shown in a previous study to be induced by flg22, a peptide representing the PAMP-active epitope of the bacterial flagella [25] (Table S4C).

### Microarray data validation by Q-RT-PCR

**Figure 4:**
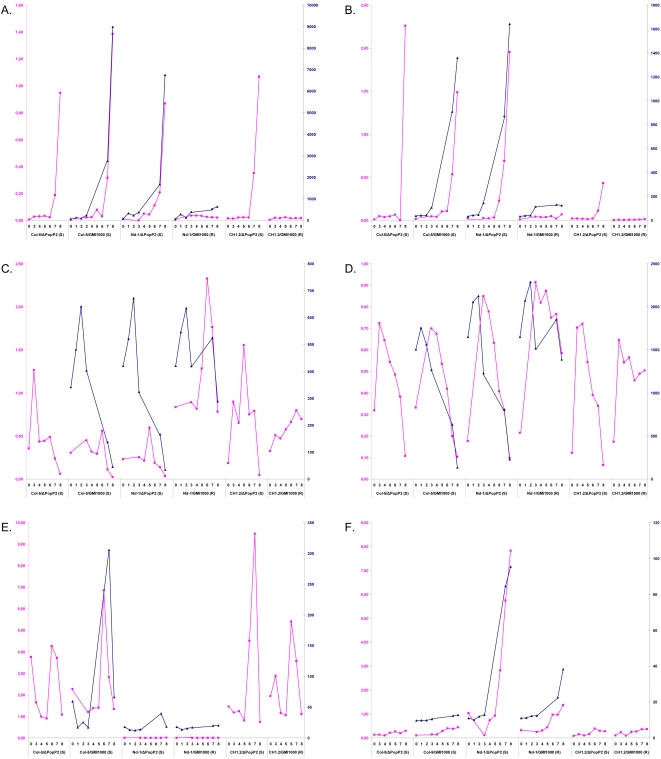
Quantitative RT-PCR validation of microarray results. The microarray data (blue lines) were validated by Q-RT-PCR (pink lines). The outcome of each interaction is indicated below each panel (R, Resistance; S, Susceptible).Samples were collected at the times indicated under each panel [0: 0H; 1: 6H; 2: 12H; 3: 24H; 4: 48H; 5: 72H; 6: 96H; 7: 120H (D1); 8: 168H (D3)] from: Nd-1 plants inoculated either with GMI1000 (R) or GMI1000ΔPopP2 (S); Col-5 plants inoculated with GMI1000 (S) or GMI1000ΔPopP2 (S); transgenic CH1.2 plants inoculated either with GMI1000 (R) or GMI1000ΔPopP2 (S). The blue scale is for normalized Affymetrix data set and the pink one is for the relative quantity of RNA by Q-RT-PCR. Each panel describes the results obtained with different genes. A. and B. Dehydrin – *Rab18* (At5g66400) and a putative Protein Phosphatase 2C (At5g59220) respectively, two genes up-regulated in diseased Nd-1 and Col-5 plants. C. and D. Basic helix-loop-helix family protein (At4g36540) and an expressed protein (At5g25460) respectively, two genes down-regulated in diseased Nd-1 and Col-5 plants. E. Ethylene-responsive element-binding protein (At4g17490), specifically up-regulated in diseased Col-5 plants. F. NADP-dependant oxydoreductase, putative (At5g16980), specifically up-regulated in diseased Nd-1 genes.

The validation of the transcriptomic data was performed by Q-RT-PCR experiments on 12 candidate genes. We evaluated the expression of these genes in different contexts. The use of CH1.2 plants, which are transgenic Col-5 plants expressing the *RRS1-R* gene, and of Nd-1 plants also expressing the *R* gene challenged with the virulent GMI1000ΔPopP2 strain allowed us to determine whether the expression of these genes during wilt disease development was affected by the genetic background. In order to detect a possible effect of the presence/absence of PopP2 on the activation of these genes, we included Col-5 plants inoculated either with strain GMI1000 or GMI1000ΔPopP2. Inoculation of both strains on this accession leads to disease development. The influence of *RRS1-R* was also studied using both Col-5 and CH1.2 plants inoculated with the virulent GMI1000ΔPopP2 strain. Plant samples were harvested at 24, 48, 72, 96 hours and 5 (D1) and 8 (D3) days after pathogen inoculation in order to check whether changes in gene expression preceded or was concomitant with wilt symptom appearance. As shown in Figure 4, (and Figure S2), Q-RT-PCR data were consistent with the microarray results. For all of the disease-activated genes tested, their expression patterns were similar, all being activated 72 or 96 hours after inoculation with activation preceding slightly or coinciding with the appearance of the first disease symptoms. None of the genes tested were induced during the incompatible interaction. Additionally, the influence of the presence of either RRS1-R or of PopP2 had no effect on the expression of the tested genes.

### Some disease-associated genes are only expressed in Nd-1

The plant genetic background had little effect on gene expression during wilt disease development. Most diseased-associated genes were indeed expressed similarly in Col-5 and Nd-1 plants and this was verified for a selected set by Q-RT-PCR. However, a search for genes specifically up-regulated only in diseased Nd-1, but not in wilting Col-5 plants, led to the identification of 172 genes whose expression was also altered in wilting Col-5 plants albeit to a lower extent, and were therefore not originally selected due to our stringent selection criteria (Figure S1B). Nevertheless, the expression of a few manually selected genes proved indeed to be altered only in the Nd-1 background in response to the GMI1000ΔPopP2 strain and remained unchanged in Col-5 plants challenged with GMI1000 (Figure 4E and Table S5A). For example, the At3g47950 gene was only activated during disease in Nd-1 plants infected with the GMI1000ΔPopP2 strain and not in CH1.2 plants challenged with the same strain, suggesting that the up-regulation of this gene was specific of the genetic background and not linked to the presence of RRS1-R (Figure S2E). Its activation was also not linked to the absence of PopP2 since no activation was detected in Col-5 plants infected with the GMI1000ΔPopP2 strain. To our knowledge, this is the first demonstration of the existence of plant genes whose expression is specifically altered during a compatible interaction in a given genetic background.

Fifty six up-regulated disease-associated “specific” Col-5 genes were also identified (Figure 4 and Table S5A). Since microarrays were generated using Col-0 probes, it is difficult to say whether these genes were specifically expressed in diseased plants in this particular accession, or are widely sequence divergent in Nd-1 plants. We favour the latter hypothesis since about 20% of these genes encode *R* or *R*-like genes that are known to evolve rapidly [26].

### Inactivation of some disease-associated genes can lead to an increased resistance to *R. solanacearum*

In order to estimate the functional importance of genes specifically expressed in compatible interactions in the process of disease development, the responses of knockout mutants for some of these genes to a virulent strain of *R. solanacearum* were tested. We tested 45 null mutants (Table S7) and most of them responded to the bacteria in a similar manner than wild type plants, which may be explained either by gene redundancy and/or by the fact that disease development requires the synergistic action of a whole battery of genes.

**Figure 5:**
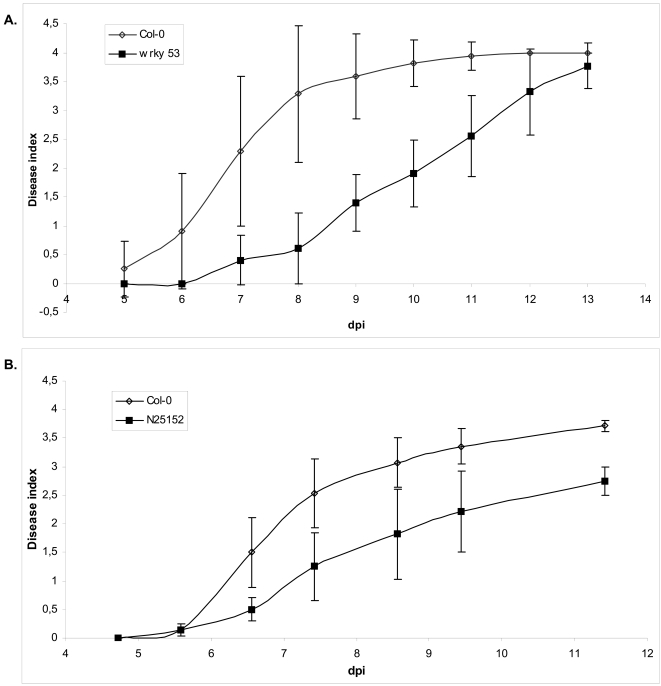
Symptom development in knockout mutant lines for WRKY53 (At4g23810), N25152 (At2g17220) and Col-0 plants. Means presented were calculated for 16 plants per plant line. Thirty mutant (filled square) and wild-type plants (open diamond) were root-inoculated with strain GMI1000 at a concentration of 10^7^ bacteria per milliliter. Plants were considered completely wilted when 100% of the rosette leaves were wilted (Disease index 4). This experiment was repeated three times with reproducible results. dpi: day post inoculation.

For some of these mutants, some altered responses were observed but not reproducibly, which suggests that environmental conditions probably play an important role in disease development. Previous data showed that plants inactivated in the *ABI1* or *ABI2*, 2 genes strongly up-regulated during wilt disease development, exhibited an increased resistance to a virulent strain of the pathogen [20]. For two additional genes encoding the transcription factor *AtWRKY53* (At4g23810) and a putative kinase (At2g17220), a significant delay in wilt symptom appearance was reproducibly observed. As shown in Figure 5, first wilt symptoms appeared in *wrky53* null plants, 2 days later than in Col-0 plants and developed slower. A similar delay was observed with the other knockout mutant (At2g17220).

## Discussion

The identification of plant targets of microbial effectors constitutes a major challenge in modern phytopathology. Effectors perturb a variety of sub-cellular and multi-cellular host defense processes. Suppression of host defenses, manipulation of the host ubiquitination machinery and transcription, alterations of vesicle trafficking and modulation of hormone signalling represent some examples of the panoply of strategies deployed by pathogens to colonize the host [11]. Surprisingly, few studies were devoted to the global changes induced in the susceptible host by a virulent pathogen, which should give important clues about the collective function of pathogen effectors.

Little is known on the mechanisms underlying *RRS1-R*-mediated resistance, a resistance that is not associated, as is often the case, with the development of HR. Consequently, it was impossible to predict at which time events determining the issue of the interaction occur, apart from the first wilt symptoms visible 5 days after pathogen inoculation in susceptible plants. In this study, no genes preferentially expressed in the early steps of the resistance response mediated by *RRS1-R* could be detected and/or validated (Table S1). The absence of direct contact between the root-inoculated pathogen and the collected leaf samples probably explains this observation. Indeed, *R. solanacearum* is a root pathogen and this study was performed on aerial parts because root tissues of plants grown in soil were not accessible and/or too damaged by infection. Although leaf inoculations by the bacteria lead to similar symptoms than root inoculation [27], we cannot exclude the possibility that differences in the expression of early responsive genes between resistant and susceptible interactions may actually be restricted to root tissue or even to some specific cell types of this organ. This hypothesis is currently being tested by analyzing the expression of some of the early candidate genes by Q-RT-PCR using root RNA derived at these early timepoints.

Transient resistance-associated gene expression occurring very rapidly (within the first 6 hours) after pathogen challenge and therefore undetectable by our approach may also account for the absence of early gene expression in resistant plants. Alternatively, the absence of symptoms such as HR-associated lesions, which are not associated with RRS1-R-mediated resistance, may explain our failure to detect *RRS1-R*-associated gene expression. Furthermore, the *RRS1-R*-mediated resistance may be dependent on genes whose expression levels were too low to be detectable using microarray analyses. Likewise, *RRS1-R* behaves as a dominant resistance gene in transgenic plants although it was genetically defined as a recessive allele [17]. The possibility that RRS1-S is a dominant negative regulator of the function of RRS1-R cannot be excluded and might then explain why no resistance-associated gene was identified by our approach. The putative transcriptional activities of the RRS1 proteins are currently under investigation and this study should provide some clues on the potential target genes of these proteins.

Our data differed from those of Tao et al [7] who detected important overlaps in plant gene expression in the early stages of incompatible interactions and in late compatible interactions using a bacterial pathogen. Extensive analyses of gene expression have so far only been performed on a limited number of plant-pathogen interactions often characterized by the development of an HR in the resistant response. Additional studies using microorganisms with different lifestyles and inducing different plant responses might reveal whether the observations of Tao et al. may or may not be generalized.

Genes associated with disease development have also been linked to various stress responses. Many changes in gene expression observed in diseased plants were ABA-related. This phytohormone is involved in the regulation of various processes, including stomatal closure, seed and bud dormancy, and physiological responses to cold, drought and salinity stress [28]. *R. solanacearum* is a vascular pathogen that promotes obstruction of the vasculature and plant wilting. It is therefore not surprising that ABA profiles are closely related to *R. solanacearum* infection profiles. The role of ABA may however be more complex. The recent identification of the *irx* mutants that are highly resistant to *R. solanacearum* suggests that the phytohormone plays a direct role in wilt disease development [20]. The *irx* mutants constitutively express a number of ABA-associated genes and it was shown that some ABA mutants (*abi1-1*, *abi2-1* and *aba1-6*) exhibit an increased susceptibility towards *R. solanacearum* [20]. During the course of this study, we tested the response of other ABA-related genes to this bacterium but could not detect differences compared to wild type plants. It appears therefore that only certain components of ABA signalling intervene in the infectious process.

Our data also support the observation that responses to many pathogens overlap substantially with senescence signalling pathways and the existence of regulators common to both programs has previously been proposed [29].

Similarly, a high proportion of genes linked to basal resistance were also strongly activated during wilt disease development (Table S4C.). Many studies showed that suppression of plant defense mechanisms constitutes one of the major functions of microbial effectors. The massive activation of genes associated to basal resistance during wilt disease is therefore intriguing: it might be explained by the probable high concentration of PAMPs associated with the elevated bacterial populations detected within wilting plants [29]. At this late stage of infection, the combined action of these defense gene products was obviously not sufficient to restrain intense bacterial colonization accompanying disease development. It would be interesting to check whether the mechanisms underlying the induction of these PAMP-activated genes during wilt disease are similar to those occurring during the establishment of basal defense.

An increasing body of evidence on plant-pathogen systems suggests that both host and parasite genotypes interact to determine symptom severity, pathogen transmission or virulence [30], [31]. In this context, we identified a few genes that were specifically activated in compatible interactions in Nd-1 but not in Col-5 plants although this differential gene expression was not correlated with differences in the responses of these 2 Arabidopsis accessions to *R. solanacearum*. Activation of these genes was independent of *PopP2*, since it was not observed in CH1.2 plants inoculated with a ΔPopP2 strain deleted of the *avr* gene, and also of *RRS1-R*, as specific gene activation occurred in Nd-1 but not in CH1.2 plants that both possess the *R* gene. There was no obvious functional relationship between the various Nd-1-specific disease genes and it is impossible to predict whether their activation results directly or indirectly from the differential effect of one or several bacterial effectors on plant gene expression. This differential impact of bacterial effectors on plant gene expression due to the genetic context highlights the importance of host variation in the determination of parasite fitness traits.

Alterations of the responses to pathogens in plants in which disease genes have been inactivated appear to be rare events. Functional redundancy probably explains most of these observations. As reported in other processes including plant resistance, disease development may also require the concerted action of a whole set of genes and the inactivation of one of them is often ineffective. The inactivation of a WRKY gene, *AtWRKY53*, led to a significant delay in wilt symptom development. This gene seems to play a regulatory role in the early events of leaf senescence [32], [33]. WRKY53 is also involved in basal resistance against the bacterial pathogen *Pseudomonas syringae* [34]. It is strongly induced in response to the bacterial PAMP flg22 [25] and may also constitute a potential target of bacterial effectors since its expression is reduced upon challenge of Arabidopsis plants with a virulent strain of *P. syringae* pv. *tomato* but is not affected in response to mutants lacking a functional type III secretion system [35], [36]. In contrast to our data, a knockout mutant of this gene was found to be more susceptible to *P. syringae* pv. *tomato* infection [34].

The elucidation of the mechanisms underlying the increased tolerance of the WRKY 53 knock-out mutant to virulent strains of *R. solanacearum* constitutes one of our major goals. Some other genes whose inactivation caused an increased resistance to a virulent *R. solanacearum* strain were previously identified. Apart from the *irx* mutants exhibiting a complete resistance to the pathogen, disease development was also retarded in *ein2.1* plants [19], in some ABA mutants [20] as well as in a putative serine-threonine kinase (this study). Whether these genes are part of a single or several signalling pathways remains an open question currently under investigation. ABA appears to play an important role in wilt disease development. In this context, quantification of this phytohormone will be realized both in the various mutants mentioned above and also during compatible and incompatible interactions with *R. solanacearum*.

## Materials and Methods

### Plant Materials and Bacterial Strains

Arabidopsis Col-5 line is a glabrous derivative of Col-0. The *R. solanacearum* strains GMI1000 is a wild-type strain originally isolated from tomato. The ΔPopP2 strain and transgenic CH1.2 lines have already been described [17], [18]. Disease resistance phenotypes were determined by root inoculation of 4-week-old plants with *R. solanacearum* strains as reported [27]. Leaf material was used for microarray analyses.

All the Arabidopsis SALK T-DNA insertion lines were obtained from the NASC (Nottingham Arabidopsis Stock Center, http://arabidopsis.info/) as homozygous lines. The WRKY 53 allele was provided by I. Somssich, and the ARR4 and ARR6 alleles by J. Kieber.

### Microarray Experiments and Analysis

Microarray analysis was performed on the aerial parts of 5 plants collected before inoculation (0 h) and 6, 12, 24 hours, 5 days or D1 (25% of wilted leaves), and 8 days or D3 (75 of wilted leaves) after bacterial inoculation. Total RNA was isolated from frozen tissues using the NucleoSpin RNAII kit (Macherey-Nagel, GmbH&Co.KG, Düren, Germany) according to the manufacturer's recommendations. RNA concentrations were determined using a NanoDrop ND-1000 spectrophotometer (NanoDrop Technologies, http://www.nanodrop.com) and RNA integrity was confirmed by Bioanalyzer 2100 electrophoresis (Agilent Technologies, http://www.agilent.com).

Probes were synthesized from RNA samples and hybridized to the Affymetrix Arabidopsis ATH1 GeneChip arrays (Affymetrix, http://www.affymetrix.com) according to the procedures provided by the manufacturer. Probes and hybridizations were performed by the Microarray platform of the IGBMC and Génopole Alsace-Lorraine (http://www-microarrays.u-strasbg.fr).

Expression measures were normalized by the Robust Multi-array Average (RMA) [37] implemented in Bioconductor packages [38]. The pairwise comparison between the two biological replicates of each compatible interaction (Col-5/GMI1000 and Nd-1/GMI1000ΔPopP2) and the two replicates of the incompatible (Nd-1/GMI1000) was performed to identify differentially expressed genes. A statistical analysis was performed with the LIMMA package using an empirical Bayes linear modelling approach [39], [40] and *p-values* were adjusted by the Benjamini and Hochberg method which controls the False Discovery Rate (FDR) [41].

Significant up-regulated and down-regulated genes were selected using an adjusted *p-value* ≤0.05 and normalized ratios (Signal Log_2_ Ratio) ≥1 or ≤−1 respectively; relative to incompatible interaction (Nd-1/GMI1000).

Functional classification of the differentially expressed genes was done on the FatiGO web-tool (http://fatigo.bioinfo.cipf.es/) [42] and lists were analyzed using Genevestigator V3 (https://www.genevestigator.ethz.ch/)[43]. Cluster analysis of timescale results were assessed with Adap_Cluster (MIN_NR_Genes = 2, s = 0.95) [44].

Additional microarray data manipulations and comparisons were assessed using Microsoft® Excel and Microsoft Access 2002 (Microsoft, Redmond, Washington, USA).

### Accession Numbers

The microarray hybridization data have been submitted to NASCArrays (reference number /NASCARRAYS-447 /, http://affymetrix.arabidopsis.info/narrays/experimentpage.pl?experimentid=447 ).

### Real-Time RT-PCR Analysis

The aerial parts of 3 plants were collected at different time points after inoculation and RNA was purified as described above. First strand cDNA synthesis was carried out with the Superscript II reverse transcriptase (Invitrogen, http://www.invitrogen.com) as described in the manufacturer's protocol using 1 µg of total RNA. cDNAs obtained were diluted 20-fold before use.

Real time PCR (RT-PCR) analysis was performed with a LightCycler (Roche, http://www.roche-applied-science.com) using the LightCycler FastStart DNA Master^Plus^ SYBR Green I kit (Roche applied Science, http://www.roche-applied-science.com). The following conditions were used: 1 cycle of 9 min at 98°C followed by 45 cycles of 5 s at 95°C, 10 s at 65°C, and 20 s at 72°C. The primer sets used in the experiments are listed in Table S6. The specificity of the amplification was systematically checked by melting curve analysis at the end of each run of real time RT-PCR. Transcript levels were normalized for each sample with the geometric mean of three selected housekeeping genes (At1g13320, At5g59810 and At1g13440) [45]. The assay included a no template control, a standard curve of four serial dilution points (in steps of 10-fold) of a standard cDNA, and each of the tested cDNA populations.

## Supporting Information

Table S1 List of up- and down-regulated genes at early time points (12H and 24H post inoculation) in Col-5/GMI1000 and in Nd-1/ΔPopP2 plants vs. Nd-1/GMI1000 plants. FDR≤0.05 and SLR≥1 for up-regulated genes or SLR≤−1 for down-regulated genes.
(0.02 MB XLS)

Table S2 List of genes up-regulated 5 days post inoculation in Col-5/GMI1000 and in Nd-1/ΔPopP2 plants vs. Nd-1/GMI1000 plants (FDR≤0.05 and SLR≥1)
(0.09 MB XLS)

Table S3 List of genes down-regulated 5 days post inoculation in Col-5/GMI1000 and in Nd-1/ΔPopP2 plants vs. Nd-1/GMI1000 plants (FDR≤0.05 and SLR≤−1)
(0.04 MB XLS)

Table S4 List of up-regulated disease genes involved in: A. the biosynthesis and signaling of ABA [21], B. genes associated with senescence [24] and C. genes induced by flg22 [25].
(0.10 MB XLS)

Table S5 List of genes specifically up-regulated 5 days post inoculation in diseased Col-5 plants (A) or in diseased Nd-1 plants (B). These genes were selected manually from the initial list of 152 (Nd-1 specific) or 56 (Col-5 specific) genes. C. List of genes down-regulated 5 days post inoculation in diseased Col-5 plants. 19 genes were manually selected from the initial list of 317.
(0.04 MB XLS)

Table S6 List of primers used for Quantitative RT-PCR experiments.
(0.02 MB XLS)

Table S7 List of Arabidopsis knockout lines tested for their response towards *R. solanacearum*. Up- and down-regulated genes 5 days post inoculation in both compatible interactions (Col-5/GMI1000 and Nd-1/ΔPopP2) versus the incompatible one (Nd-1/GMI1000) (A and B, respectively).
(0.03 MB XLS)

Figure S1 Cluster Analysis of Col-5 and Nd-1 specific gene lists. Cluster analysis with Adap_Cluster (Min_NR_Genes = 2, s = 0.95). A. 166 up-regulated genes assigned to the first cluster and 93 up-regulated genes assigned to the second cluster of the Col-5 specific gene list. B. 132 up-regulated genes assigned to the first cluster of the Nd-1 specific gene list. For each cluster, the mean expression profile is shown in red.
(9.20 MB TIF)

Figure S2 Quantitative RT-PCR validation of selected differentially expressed genes at different time points. The microarray data (blue lines) were validated by Q-RT-PCR (pink lines). Samples were collected at the times indicated under each panel [0: 0H; 1: 6H; 2: 12H; 3: 24H; 4: 48H; 5: 72H; 6: 96H; 7: 120H (D1); 8: 168H (D3)] from: Nd-1 plants inoculated either with GMI1000 (R) or GMI1000ΔPopP2 (S); Col-5 plants inoculated with GMI1000 (S) or GMI1000ΔPopP2 (S); transgenic CH1.2 plants inoculated either with GMI1000 (R) or GMI1000ΔPopP2 (S). The outcome of each interaction is shown below each panel (R, Resistance; S, Susceptible). The blue scale is for normalized Affymetrix data set and the pink one is for the relative quantity of RNA by Q-RT-PCR. A., B., C. and D. Protein phosphatase 2C, *ABI2* (At5g57050), Protein Phosphatase 2C, *ABI1* (At4g26080), 9-cis-epoxycarotenoid dioxygenase, putative (At3g14440), Rieske (2Fe-2S) domain-containing protein (At3g44880); 4 up-regulated genes in diseased Nd-1 and Col-5 plants. E. ATPase, plasma membrane-type, putative (At3g47950), a gene specifically up-regulated in diseased Nd-1. F. Peroxidase-related (At5g51890), a gene specifically down-regulated in resistant Nd-1 and CH1.2 plants.
(4.47 MB TIF)
