# Supplementary figures and images for: Transcriptional Responses of Arabidopsis thaliana during Wilt Disease Caused by the Soil-Borne Phytopathogenic Bacterium, Ralstonia solanacearum
Source: PLoS One. 2008 Jul 2;3(7):e2589. doi: 10.1371/journal.pone.0002589 (PMC2435627; doi:10.1371/journal.pone.0002589)

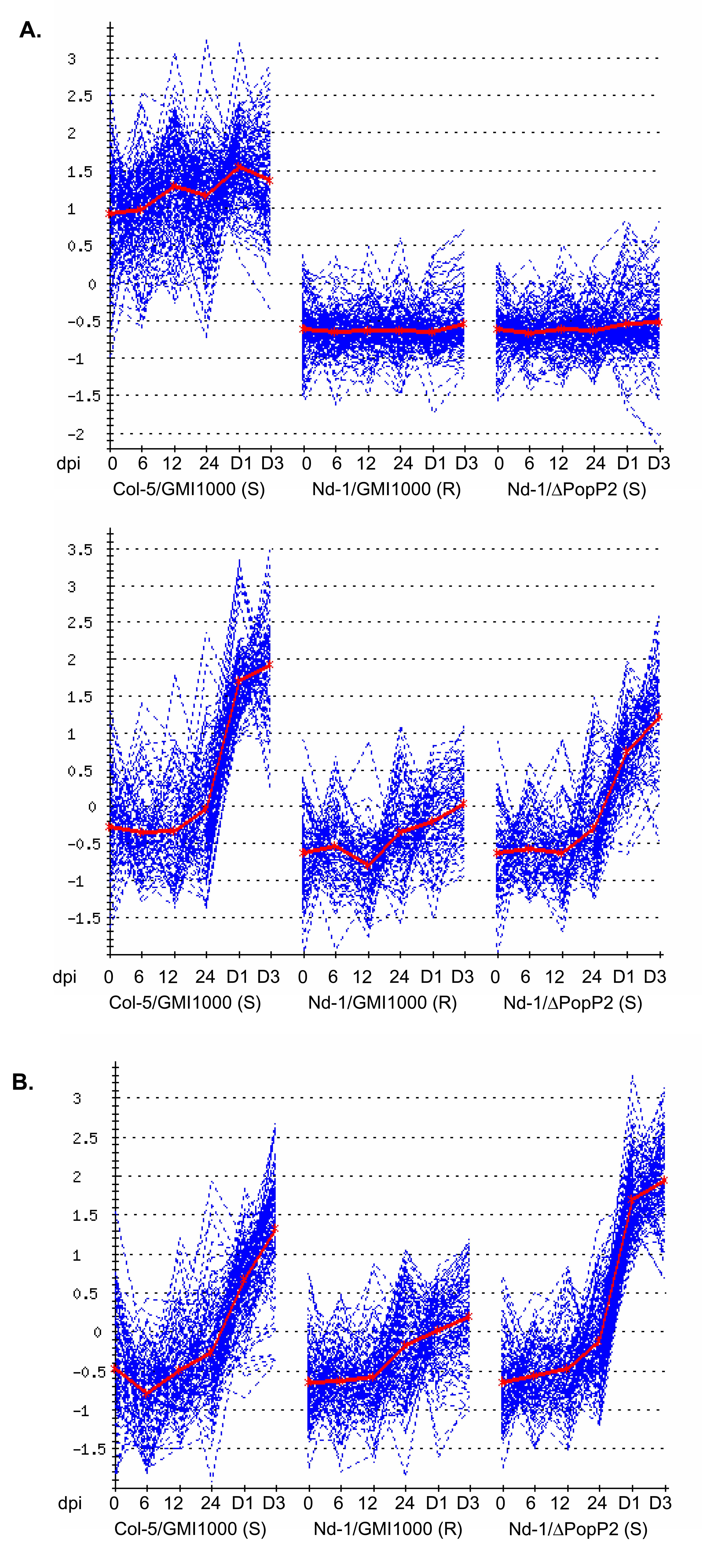

Supplement: Figure S1 — Cluster Analysis of Col-5 and Nd-1 specific gene lists. Cluster analysis with Adap_Cluster (Min_NR_Genes = 2, s = 0.95). A. 166 up-regulated genes assigned to the first cluster and 93 up-regulated genes assigned to the second cluster of the Col-5 specific gene list. B. 132 up-regulated genes assigned to the first cluster of the Nd-1 specific gene list. For each cluster, the mean expression profile is shown in red. (9.20 MB TIF) [file pone.0002589.s008.tif]

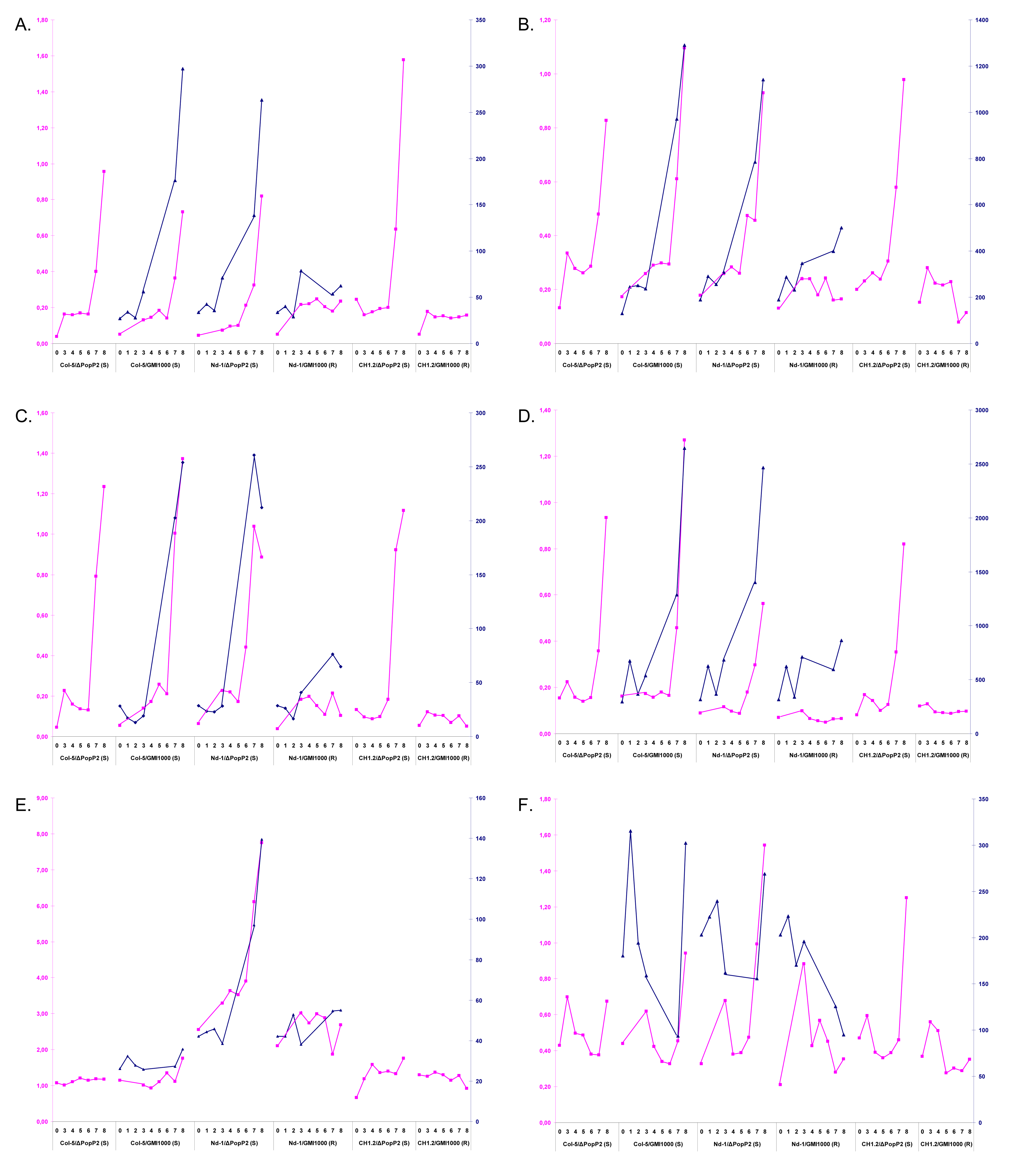

Supplement: Figure S2 — Quantitative RT-PCR validation of selected differentially expressed genes at different time points. The microarray data (blue lines) were validated by Q-RT-PCR (pink lines). Samples were collected at the times indicated under each panel [0: 0H; 1: 6H; 2: 12H; 3: 24H; 4: 48H; 5: 72H; 6: 96H; 7: 120H (D1); 8: 168H (D3)] from: Nd-1 plants inoculated either with GMI1000 (R) or GMI1000ΔPopP2 (S); Col-5 plants inoculated with GMI1000 (S) or GMI1000ΔPopP2 (S); transgenic CH1.2 plants inoculated either with GMI1000 (R) or GMI1000ΔPopP2 (S). The outcome of each interaction is shown below each panel (R, Resistance; S, Susceptible). The blue scale is for normalized Affymetrix data set and the pink one is for the relative quantity of RNA by Q-RT-PCR. A., B., C. and D. Protein phosphatase 2C, ABI2 (At5g57050), Protein Phosphatase 2C, ABI1 (At4g26080), 9-cis-epoxycarotenoid dioxygenase, putative (At3g14440), Rieske (2Fe-2S) domain-containing protein (At3g44880); 4 up-regulated genes in diseased Nd-1 and Col-5 plants. E. ATPase, plasma membrane-type, putative (At3g47950), a gene specifically up-regulated in diseased Nd-1. F. Peroxidase-related (At5g51890), a gene specifically down-regulated in resistant Nd-1 and CH1.2 plants. (4.47 MB TIF) [file pone.0002589.s009.tif]
